# Complete Genome Sequence Analysis of a Reassortant Strain of Bluetongue Virus Serotype 16 from Italy

**DOI:** 10.1128/genomeA.00622-13

**Published:** 2013-08-22

**Authors:** Alessio Lorusso, Adalberto Costessi, Walter Pirovano, Maurilia Marcacci, Cesare Cammà, Giovanni Savini

**Affiliations:** OIE Reference Laboratory for Bluetongue, Istituto Zooprofilattico Sperimentale dell’Abruzzo e Molise G. Caporale, Teramo, Italya; BaseClear BV, Leiden, The Netherlandsb

## Abstract

The complete genome sequence of a reassortant field strain of bluetongue virus serotype 16 (BTV-16), isolated from cattle in the Apulia region of Italy in 2002, has been determined by Illumina sequencing. Sequence comparisons of segment 1 (Seg-1) to Seg-10, except Seg-5, show that BTV-16 strain ITL2002 belongs to the major eastern topotype of BTV.

## GENOME ANNOUNCEMENT

The occurrence of mutation, insertion/deletion, and reassortment/recombination events in RNA viruses means that each infected host is likely to carry viral populations with potentially high genetic diversity. Rapid retrieval of complete and accurate genomic data is fundamental in order to elucidate the emergence and molecular epidemiology of these viruses. A full-genome approach becomes crucial when dealing with segmented RNA viruses, such as influenza A virus (1, 2) or bluetongue virus (BTV) (3), or with viruses with extraordinarily plastic genomes, such as coronaviruses (4, 5, 6). BTV is the prototype of the genus *Orbivirus*, within the family *Reoviridae* (7), and causes bluetongue, a disease affecting wild and domestic ruminants (8). BTV includes 26 distinct serotypes (7, 9, 10) and its genome is composed of 10 segments (Seg-1 to Seg-10) of linear double-stranded RNA (dsRNA) (11). Total dsRNA (12) was used as input for library preparation using the Illumina TruSeq RNA library preparation kit (Illumina) with modifications. dsRNA was fragmented and subjected to first-strand cDNA synthesis (13). After second-strand synthesis, bar-coded DNA adapters were ligated to both ends of the double-stranded cDNA, and the ligated product was size selected and subjected to PCR amplification. The resultant library was checked on a Bioanalyzer (Agilent) and quantified. The libraries were multiplexed, clustered, and sequenced on an Illumina HiSeq 2500 genome analyzer with a paired-end protocol. The sequencing run was analyzed with the Illumina CASAVA pipeline (v 1.8.3), with demultiplexing based on sample-specific bar codes, obtaining 260 Mb of sequence data. The quality of the sequences was enhanced by trimming off low-quality bases using the CLC Genomics Workbench. The quality-filtered sequence reads were aligned against the reference sequences by use of the CLC Genomics Workbench. The 10 segments of BTV serotype 16 (BTV-16) strain ITL2002 were amplified successfully for their entire length (14). Sizes in base pairs for Seg-1 to Seg-10 of BTV-16 ITL2002 were 3,944, 2,935, 2,272, 1,981, 1,776, 1,637, 1,156, 1,125, 1,052, and 822, respectively. BTV-16 was isolated for the first time in 1960 in Pakistan, supplied to the Onderstepoort Veterinary Research Institute, and named RSArrrr/16 (South Africa). It was recently fully sequenced at Pirbright, United Kingdom (14). All segments except Seg-5 showed >99% sequence identity with RSArrrr/16 and the corresponding vaccine strain RSAvvvv/16. Equal levels of identity were demonstrated with the Chinese BTV-16 (strain BN96/16) isolated from a sheep in 1996. Seg-5 of BTV-16 ITL2002 is identical to that of the BTV-2 vaccine strain (RSAvvvv/02). Indeed, BTV-16 ITL2002 Seg-5 shows only ~82% to 83% nucleotide identity with the other BTV-16 strains, suggesting that this segment is likely derived from genomic reassortment between RSAvvvv/16 and RSAvvvv/02, widely used in the Mediterranean basin in the past (15). Multiple BTV serotypes are currently circulating in the southern Mediterranean basin (4), thus potentially leading to the spreading of novel reassortant viruses with unpredictable biological features. Full-genome sequencing and sharing of genomic data are therefore highly recommended in order to promptly identify the emergence of viruses with novel genome segment constellation.

### Nucleotide sequence accession numbers. 

Nucleotide sequences for BTV-16 ITL2002 have been deposited in GenBank with the accession numbers KF387521 to KF387530.
